# Acclimatisation to tropical seasons: hydric and thermal physiology in *Gehyra* geckos

**DOI:** 10.1242/jeb.250797

**Published:** 2026-03-13

**Authors:** Kade N. Skelton, Craig C. Moritz, Kimberley A. Day, Chava L. Weitzman, Christine A. Schlesinger, Stephen M. Zozaya, Keith A. Christian

**Affiliations:** ^1^ Charles Darwin University, Research Institute for the Environment and Livelihoods, Ellengowan Drive, Brinkin, NT 0810, Australia; ^2^ Division of Ecology and Evolution, Research School of Biology, and Centre for Biodiversity Analysis, The Australian National University, Building 116, Daley Road, Acton, ACT 2601, Australia

**Keywords:** Acclimatisation, Evaporative water loss, Reptiles, Seasonal physiology, Seasonal tropics, Thermal preference

## Abstract

Seasonal acclimatisation is a mechanism enabling individuals to advantageously adjust one or more physiological parameters in response to changing environmental conditions. The ability to adjust metabolic rates and thermal physiology in response to seasonal changes is known to be central to the physiological ecology of some reptiles, but few studies have examined the ability of reptiles to exhibit seasonal flexibility in rates of evaporative water loss (EWL). We measured acclimatisation to seasonal changes for both temperature and water-related traits in six species of geckos in the genus *Gehyra* from the highly seasonal tropics of northern Australia. Four species from a mesic, more thermally stable site did not have seasonal differences in thermal preference (*T*_pref_), but *T*_pref_ was significantly lower during the cooler dry season in three species from a semi-arid, more thermally variable site. EWL was lower (34–76% reduction) during the dry season compared with the wet season, representing a significant reduction for all gecko species. EWL decreased rapidly from wet to early dry season, then either remained low or continued to decrease to a minimum in the late dry season. These results indicate acclimatisation in EWL, resulting in the conservation of water during the dry season. A growing body of evidence suggests that seasonal acclimatisation of EWL broadly occurs in lizards in the wet–dry tropics of Australia, but less is known about seasonal acclimatisation of EWL in other geographic regions.

## Teaser

**Summary:** Six gecko species in the seasonal tropics of Australia reduce their rates of water loss during the dry season, but thermal preference is only responsive in the more thermally variable site.

## INTRODUCTION

Seasonal fluctuations in environmental conditions can expose ectothermic animals to periods of unfavourable conditions that can present challenges to survival, such as prolonged dry periods when moisture is limiting, or extreme temperatures (Christian et al., 1996; Nagy, 1972). Physiological acclimatisation, the ability to adjust physiological responses to compensate for environmental changes, can enable animals to persist during temporarily adverse conditions or cope with a change in climate factors, such as environmental temperature (Basson and Clusella-Trullas, 2015; Bozinovic and Naya, 2014; Dubiner et al., 2023), reduced food availability (Canale and Henry, 2010; Christian et al., 1999a) and water availability (Blamires and Christian et al., 1999; Day et al., 2025). A greater capacity for acclimatisation increases tolerance to a broader range of environmental conditions, and the lack of acclimatisation could restrict activity times or decrease survival (Bozinovic and Naya, 2014; Muñoz and Bodensteiner, 2019). Therefore, a greater capacity for physiological acclimatisation is expected in species from more variable climates (Christian et al., 2024; Muñoz and Bodensteiner, 2019; Sheldon et al., 2018). It is sometimes assumed that the relatively stable climatic conditions of tropical environments limit the potential for physiological acclimatisation (Sun et al., 2022). However, animals from the wet–dry tropics experience significant seasonal differences in humidity and food and water availability, despite relatively stable environmental temperatures (Christian et al., 1999a, 2024).

Reptiles can modify a variety of behavioural and physiological traits across seasons in response to changed abiotic variables (Christian et al., 1998, 2024; Clusella-Trullas and Chown, 2014; Seebacher et al., 2015; Somero, 2010). In some reptiles, preferred body temperatures change seasonally as measured in the field or measured from animals immediately after capture, with lower body temperatures selected during the dry season to conserve energy and water when food and water are scarce (Angilletta, 2009; Christian et al., 1983, 1998; Christian and Bedford, 1995, 1996; Clusella-Trullas and Chown, 2014). Reduced metabolic rates during seasons when food and water are limiting can allow animals to survive these predictable periods of adverse conditions (Berg et al., 2017; Christian et al., 1999a; Dubiner et al., 2023; Hailey and Loveridge, 1997; Guppy and Withers, 1999). A detailed seasonal energy balance of frillneck lizards (*Chlamydosaurus kingii*) demonstrated that such metabolic depression was critical to their survival during the dry season with decreased food availability (Christian et al., 1996). The relatively high environmental temperatures in their tropical environment results in disproportionately large energy savings because of the non-linear relationship between metabolic rate and temperature (Christian et al., 1999a,b; Berg et al., 2017).

The control of water loss, including by way of evaporation, is a fundamental aspect of terrestrial life and reptile ecology (Mautz, 1982; Pirtle et al., 2019). Evaporative water loss (EWL) is an important component of hydric physiology for terrestrial reptiles challenged with maintaining hydration in environments where dehydration is a risk. Although total EWL consists of respiratory, ocular, cloacal and cutaneous EWL, cutaneous EWL accounts for most of the non-excretory water loss in reptiles (Bentley and Schmidt-Nielsen, 1966; Mautz, 1982). In a study of an arid zone gecko in the genus *Gehyra*, the availability of water was more limiting on individual traits and population structure than was high temperature (Grimm-Seyfarth et al., 2018). On an evolutionary timescale, water availability and EWL were deemed more constraining than the thermal environment in the relictual Mediterranean lizards of the genus *Algyroides* (Carneiro et al., 2017). Although reptiles from arid habitats often have lower rates of EWL than those from more mesic climates (Belasen et al., 2017; Bentley and Schmidt-Nielsen, 1966; Cox and Cox, 2015; Dmi'el, 2001; Mautz, 1982; but see Skelton et al., 2025), less is known about seasonal evaporative water loss in reptiles compared with seasonal thermal and metabolic physiology (Rozen-Rechels et al., 2019a,b; Weaver et al., 2023). The available information suggests a trend for lower EWL in reptiles in dry seasons compared with wet seasons (Blamires and Christian, 1999; Day et al., 2025).

Just as temperature and metabolic rate are intimately linked in ectothermic animals, temperature and hydric physiology interact in critical ways that can be complex (Rozen-Rechels et al., 2021). Recent studies have shown that dehydration induces water conservation in ways that tend to compromise thermoregulatory patterns (Kearney et al., 2018; Le Galliard et al., 2021a,b; Rozen-Rechels et al., 2019a,b, 2020; Sannolo and Carretero, 2019), but the selection of humid microhabitats can ameliorate the consequences of dehydration to some extent (Bodineau et al., 2024). Similarly, the nocturnality of geckos exposes them to cooler and less dehydrating microclimatic conditions than those experienced by diurnal reptiles.

Recent work on *Gehyra* geckos has revealed new species and crypic lineage diversity as well as phenotypic divergence associated with the use of different microhabitats. Some species have restricted ranges, often associated with mesic rocky habitat, while others are widespread and inhabit the surrounding seasonally arid savanna (Moritz et al., 2018; Oliver et al., 2019, 2020). In addition to providing information about this diverse genus, investigating the physiology of *Gehyra* geckos addresses broader questions about the relationship between physiology, season, and climate in tropical reptiles. By exploring the hydric and thermal physiology of *Gehyra* geckos, this study investigates how species from seasonal tropical climates respond to environmental variability, contributing to a broader understanding of reptile ecophysiology and addresses the assumption that tropical species have a lower capacity for physiological plasticity than temperate reptiles (Christian et al., 2024).

**Fig. 1.**
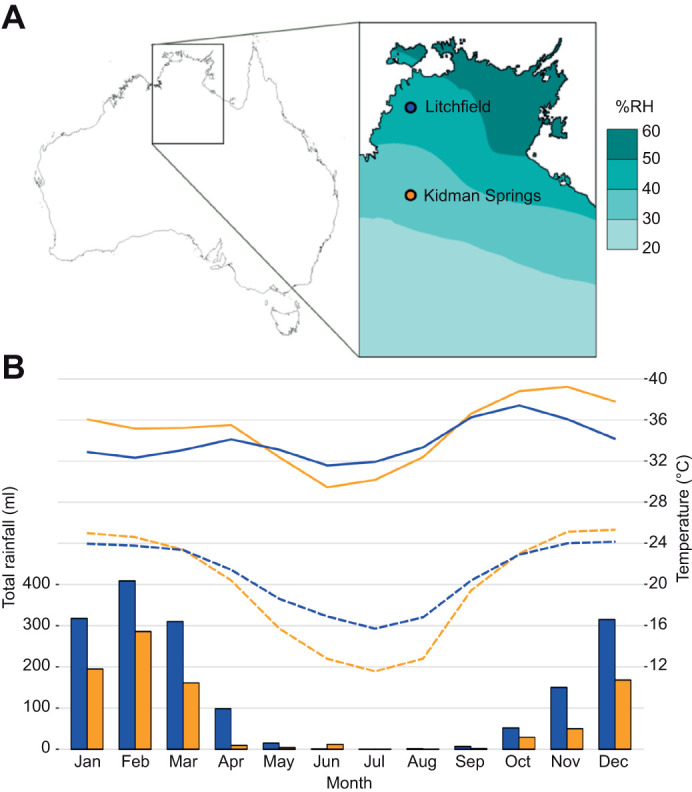
**Study sites from which *Gehyra* gecko species were sampled for physiological acclimatisation studies.** (A) Map of the northern portion of the Northern Territory displaying sampling locations. Colour overlay, provided for context, represents mean annual 15:00 h relative humidity (% RH) between 1976 and 2005. Figure modified from map provided by the Australian Bureau of Meteorology, http://www.bom.gov.au/climate. (B) Mean monthly total rainfall, and mean maximum (solid lines) and minimum temperatures (dashed lines), for the two sites from 2000 to 2009 inclusive. Litchfield data (blue) were sourced from the Batchelor Airport weather station and Kidman Springs data (orange) were sourced from the Kidman Springs weather station.

We investigated seasonal acclimatisation in thermal preferences (*T*_pref_) and EWL of nocturnal *Gehyra* geckos from the seasonal tropics of northern Australia. Body temperature is a fundamental aspect of reptile ecology that indirectly influences fitness by directly influencing ecological and physiological activities (Huey, 1982). Geckos were sampled from two sites where they are active year-round, and although the two sites differ in aridity and seasonal temperature patterns, both are located in the seasonal tropics of northern Australia. Rainfall and humidity differ between wet and dry seasons across the region in association with the summer monsoon, and even relatively arid sites are humid during wet seasons (Fig. 1). Based on previous work on lizards from the wet–dry tropics of Australia, we hypothesized that: (1) geckos have the physiological capacity to adjust EWL seasonally (Blamires and Christian, 1999; Day et al., 2025) to conserve water resources in the dry season, and (2) *T*_pref_ would be higher in wet season (warm) than the dry (cool) season (Christian et al., 1998) to conserve energy and water resources in the dry season.

## MATERIALS AND METHODS

### Species and sites

The Parks and Wildlife Commission of the Northern Territory provided permits (64816, 66691, 69132), and the work was approved by the Charles Darwin University's Animal Ethics Committee (A19005). Six species of *Gehyra* Gray 1834 were sampled from two sites [Litchfield National Park (−13.12 latitude, 130.80 longitude) and Kidman Springs Station (−16.12, 130.96); Fig. 1A], both with strong seasonal changes in rainfall (Fig. 1). Litchfield has mesic conditions (mean annual rainfall, 1675 mm) with low seasonal fluctuation in temperature, and Kidman Springs is semi-arid (mean annual rainfall, 914 mm) with greater seasonal changes in temperature (Fig. 1B, based on data averaged over 2000–2009 inclusive, acquired from the Australian Bureau of Meteorology, http://www.bom.gov.au/climate). Although the two sites represent distinct climates within the seasonal tropics, we acknowledge that using a single location per climate type may not fully capture the variability or be entirely representative of the broader effects of climatic seasonality on seasonal physiological responses.

Because of constraints of geographic range, each species was sampled from a single location except for the geographically widespread *Gehyra nana*, which was sampled from both locations (Table 1). The species identity of all individuals was confirmed by sequencing of mtDNA, which provides a reliable diagnostic for these taxa, some of which are difficult to distinguish based on morphology (Oliver et al., 2020).

**Table 1.** Species and locations of *Gehyra* geckos sampled in 2019–2021 for experiments comparing evaporative water loss (EWL) and thermal preference (*T*_pref_) among seasons

Species	Sampling location(s)	Season	EWL (*n*)	*T*_pref_ (*n*)
*Gehyra australis*	Litchfield (12 female, 16 male, 7 unknown)	Wet	13	12
		Early dry	10	0
		Dry	12	5
*Gehyra gemina*	Kidman Springs (15 female, 11 male, 1 unknown)	Wet	11	12
		Early dry	8	0
		Dry	8	11
*Gehyra koira*	Kidman Springs (12 female, 13 male)	Wet	11	12
		Early dry	5	0
		Dry	9	11
*Gehyra lapistola*	Litchfield (21 female, 19 male, 1 subadult, 11 unknown)	Wet	12	13
		Early dry	8	0
		Dry	32	20
*Gehyra nana*	Kidman Springs (17 female, 14 male)	Wet	9	12
		Early dry	12	0
		Dry	10	12
	Litchfield (20 female, 13 male, 6 subadult, 6 unknown)	Wet	15	15
		Early dry	8	0
		Dry	22	14
*Gehyra paranana*	Litchfield (19 female, 7 male, 2 subadult, 10 unknown)	Wet	11	11
		Early dry	10	0
		Dry	17	7

Geckos were sampled during the wet (November–February), early dry (May–June) and late dry (referred to as ‘dry’, July–September) seasons (Table 1). They were located in the field by spotlight, captured by hand, and adult individuals were transported to Charles Darwin University in cloth bags. To ensure that measurements reflected the conditions of the animals in the field rather than acclimation to laboratory conditions, EWL was measured in the laboratory over the following 24 h, followed by *T*_pref_ experiments over the next 3 days. After the experiments, geckos were sexed and measured using digital calipers and an electronic balance (Table S1) and housed individually in clear plastic cages (40×25×13.5 cm) containing a plastic hide in a temperature-controlled room at 28°C with 12 h:12 h light:dark, and they were supplied with a spray of clean water daily and live food (one or two mealworms and one or two baby crickets) three times per week until release at the site of capture.

### Thermal preference

Thermal preference was measured using the techniques of Day et al. (2025). Geckos were individually placed in a glass tank (modified aquarium, 59×34×37 cm) with an artificial crevice made from a 54×15×0.8 cm length of ceramic tile elevated 1.5 cm by terracotta blocks. The tank was housed in a temperature-controlled room set to an air temperature of 19.5°C and a 50 W infrared heat globe (One Reptile, Kong's Pty Ltd., Ingleburn, NSW, Australia) was placed at one end of the crevice to create a non-linear substrate temperature gradient of ∼20–40°C (Belasen et al., 2017; Christian et al., 1998; Carneiro et al., 2017; Rozen-Rechels et al., 2020). Each gecko was in the thermal gradient tank for 48 h, allowing an overnight period for the animals to settle. The animals had not fed for at least 12 h before the beginning of the experiment, and they were not fed during the experiment. Thermal images were manually collected (from a distance of 20–30 cm) during the day over the following day and a half at hourly intervals for a total of 12 measurements per gecko. A thermal imaging camera (Testo 868, 0.08°C thermal sensitivity) was used to measure surface temperatures of the geckos, and we extracted temperatures using Testo IRSoft thermal imaging software (v.4.8) from the lower abdomen of the animal (Fig. A4 in Skelton et al., 2025). The mean *T*_pref_ was calculated from the central 50% of readings (the set-point range, Hertz et al., 1993; Pintor et al., 2016; Stellatelli et al., 2018).

### Evaporative water loss

Although EWL includes components due to evaporation across the skin, from the eyes and from respiratory water loss (Mautz, 1982), we measured the total EWL as the ecologically relevant metric. EWL was measured with an open-flow system (Day et al., 2025; Mautz, 1982; Young et al., 2005) during the day. Air was drawn through silica gel drying columns and into cylindrical experimental chambers (13.5×2.6 cm, 70 ml volume) at a rate of 0.2 l min^−1^ using low-flow air pumps (Sensidyne Gilian LFS-113D) calibrated using a Gilian primary flow calibrator (Gilibrator 2, Synsidyne). These chambers were housed in an AandE Lab 18 l portable incubator (model AE-PI-100) set to 30°C. Vaisala HUMICAP^®^ Humidity and Temperature Probes HMP110 (0–100±1.5% RH, −40–80±0.1°C) were also housed in the incubator downstream of the animal chamber, and they were connected to a data acquisition system (ADInstruments PowerLab, model PL3508, paired with LabChart software, ADInstruments Pty Ltd, Bella Vista, Australia) to continuously record temperature and relative humidity of the air exiting the chambers. Five parallel systems were housed in the incubator to allow simultaneous measurements from multiple animals, and the chamber ID was included as a random effect in the analyses described below. The experimental temperature of 30°C represents an ecologically relevant temperature experienced by these geckos (Skelton et al., 2025) and has been used in similar studies (Day et al., 2025; Skelton et al., 2025). Baseline measurements were taken from stable readings before an animal was introduced in the chamber, and the baseline was confirmed at the end of each measurement. Mean baseline air temperature was 30.4°C (±1.8°C), and mean relative humidity was 8.5% (±2.4%; mean vapour pressure=0.37 kPa±0.11 kPa; mean vapour pressure deficit=4.00±0.45 kPa).

After a gecko was placed in the chamber, temperature and relative humidity of the air were monitored until readings stabilized and the animal remained at rest for at least 10 min. The humidity sensors responded to even slight movements, thus resting periods were easily determined by inspection of the electronic trace. The lowest humidity readings over a 2 min period during this rest period were used in the analyses. Trials lasted no longer than 2 h. The readings were very stable while the animals were at rest, but if an animal defecated or failed to rest during the experiment, it was re-run later that day. The difference between the reading with the animal in the chamber and the baseline is a measure of the amount of water lost from the animal and was calculated from the equations of Bernstein et al. (1977) for an open-flow system using saturation vapour density (to determine the mass of water from measurements of relative humidity) calculated from the equations of List (1971).

Evaporative water loss was expressed as the total mass flow of water from the animal (*M*_w)_, as calculated by:
(1)

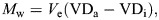
where *V*_e_ is experimental flow rate, VD_a_ is water vapour density of the air in the experimental chamber with the animal (g cm^−3^) and VD_i_ is baseline water vapour density (g cm^−3^).

Thus, water loss measurements were calculated relative to baselines, and the humidity sensors were periodically calibrated against known humidities above saturated salt solutions in a Vaisala (Helsinki) Humidity Meter Calibrator, model HMK11. Total water loss was analysed with respect to body surface area (Mautz, 1982; see below), which was estimated for each individual based on its mass using the *TrenchR* R package (https://CRAN.R-project.org/package=TrenchR; Buckley et al., 2023).

### Statistical analysis

Analyses were performed with R v.4.4.0 in RStudio v.2024.4.2.7 (r-project.org). We used Bayesian mixed-effects models implemented in MCMCglmm v.2.36 (https://CRAN.R-project.org/package=MCMCglmm; Hadfield, 2010) to test the effects of season on body condition, *T*_pref_ and EWL. For each trait, we first fitted a phylogenetic regression to estimate general seasonal trends among *Gehyra* spp. while accounting for shared evolutionary history, followed by a non-phylogenetic regression that included a species×season interaction to assess among-species variation in seasonal responses. Models incorporating phylogenetic covariance were based on the phylogeny of Lau et al. (2025), pruned to include only the taxa sampled in this study. Because *G. nana* represents an unresolved species complex with substantial within-lineage geographic structure (Moritz et al., 2018; Read et al., 2025), we treated the two sampled populations of *G. nana* as separate species for analysis by adding an extra tip to the phylogeny using the bind.tip function in phytools, placed as sister to the *nana2* tip with shallow divergence (node height=0.0197768). For relevant analyses, the phylogenetic covariance matrix was calculated using the inverseA function in MCMCglmm; parameter-expanded priors were specified for the phylogenetic random effect (*V*=1; ν=1; α.μ=0; α.*V*=1000). For all analyses, we used weakly informative inverse-gamma priors on the residual variance (*V*=1; ν=0.002) and specified the family as ‘Gaussian’. Markov chain Monte Carlo (MCMC) sampling was run for 420,000 iterations, with a burn-in of 20,000, and thinning interval of 100, yielding 4000 posterior samples. Convergence and effective sample sizes were verified from trace plots, autocorrelation diagnostics, and effective sample size estimates. For each fixed effect, we report posterior means, 95% credible intervals (CI), and two-sided pMCMC values (calculated as twice the proportion of posterior draws opposite in sign to the mean estimate). Pairwise seasonal contrasts were calculated from posterior draws of the linear predictor and visualised using forest plots. To correct for multiple comparisons, we applied a false discovery rate (FDR) adjustment – defined as the expected proportion of false positives among rejected hypotheses across repeated tests (Benjamini and Hochberg, 1995) – to all pMCMC values for a given response variable. To aid interpretation and visualise among-species variation, we generated posterior predictions for each species in each season for both *T*_pref_ and EWL, at mean values of surface area (for EWL) and body condition, averaged across sex.

While not a primary focus of this study, we assessed the effect of season on body condition, as condition may influence physiological traits such as *T*_pref_ and EWL. These analyses used the EWL dataset, which provided a larger sample size and included individuals from the wet, early dry, and dry seasons. In the phylogenetically informed model, log-transformed body mass was the response variable, with log-transformed snout–vent length (SVL) and season included as fixed effects. A second model, excluding phylogenetic covariance, included a species×season interaction to test for among-species variation in seasonal effects. Because body condition varied seasonally in some species, we incorporated it into the analyses of *T*_pref_ and EWL. To do this, we created a body condition variable by taking the residuals from a linear regression of log(mass) on log(SVL) using the lm function in R.

The phylogenetically informed analyses of preferred body temperature included *T*_pref_ as the response variable, with sex, body condition, and season (wet and dry) as fixed effects, and phylogenetic covariance as a random effect. A second model excluded the phylogenetic random effect but incorporated a species×season interaction to assess among-species variation in seasonal responses.

Analyses of EWL data required accounting for surface area (SA) and missing values for sex. SA was included to control for body size-related variation in EWL and was calculated from body mass (Fei et al., 2012) using the surface_area_from_mass function in *TrenchR* v.1.1.1 (Buckley et al., 2023; Skelton et al., 2025), specifying ‘lizard’ as the taxon. For all analyses, EWL was log-transformed to accommodate the relationship between mean and variance with respect to SA. Missing sex values were present for 44 of 253 individuals and were addressed using multiple imputation with mice v.3.16.0 (van Buuren and Groothuis-Oudshoorn, 2011). Following the rationale outlined by Blomberg and Todorov (2025), we emphasise that the goal of multiple imputation is not to recover the ‘true’ sex of unknown individuals, but to propagate uncertainty associated with missing values through the analysis. Accordingly, we generated multiple imputed datasets and pooled posterior estimates across imputations, rather than treating imputed values as observed data. This approach avoids the known biases and overconfidence associated with single imputation while retaining statistical power. We used a two-level logistic model with a random intercept for species and predictors including log(EWL), SA, body condition, season, chamber and species. Thirty imputed datasets were generated, ensuring consistent factor levels across imputations. For each imputed dataset, we fitted a phylogenetically informed model of EWL, with EWL as the response variable, SA, sex, body condition and season (wet, early dry, dry) as fixed effects, and phylogenetic covariance and chamber as random effects. A second model excluded the phylogenetic random effect but incorporated a species×season interaction to assess among-species variation in seasonal responses. Posterior samples from all imputations were concatenated to generate pooled posterior estimates. Statistical significance was considered at pMCMC<0.05.

## RESULTS

There was a significant effect of season on body condition in the phylogenetically informed analysis, accounting for the significant relationship between log(mass) and log(SVL); however, the effect of season was not robust to FDR adjustment for multiple comparisons (Table S3). In the species×season analysis, a seasonal effect on body condition was evident in the three species sampled at Kidman Springs (*Gehyra gemina*, *Gehyra koira* and *G. nana* [KS]), with geckos being heavier than expected for a given SVL in the wet season compared with the dry (Table S4). This relationship remained significant after FDR adjustment for *G. gemina* and *G. nana*, but not for *G. koira*, although estimates suggested a similar trend. The weaker support for *G. koira* may reflect limited statistical power (i.e. insufficient sample size) rather than biological inconsistency, as all three species at Kidman Springs showed broadly consistent patterns in a site that is harsher and more variable than Litchfield.

### Thermal preference

**Fig. 2.**
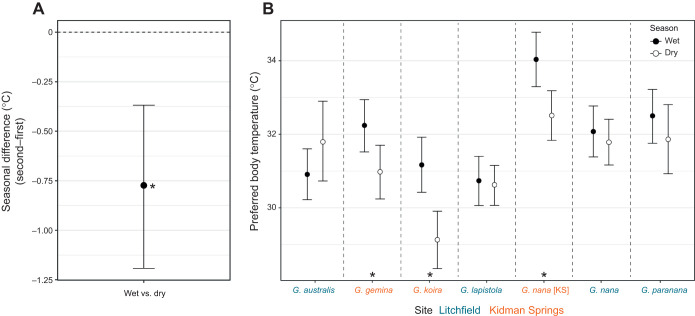
**Seasonal variation in preferred body temperature (*T*_pref_).** (A) Pairwise contrast of *T*_pref_ between the wet and dry seasons (posterior mean±95% CI), showing lower *T*_pref_ in the dry season. (B) Predicted *T*_pref_ values for each species by season, showing posterior mean and 95% CI. Coloured text indicates the two sampling sites: the more mesic Litchfield National Park and the more arid Kidman Springs. Asterisks denote species with significant seasonal differences at *P*<0.05. All values and effect sizes are adjusted to the mean body condition and averaged across sex. See Table 1 for sample size for each species.

Preferred body temperature was significantly correlated with both sex and season, but not with body condition (Table S5), and results were robust to FDR adjustment. Males preferred temperatures on average 0.57°C lower than females (95% CI=0.16–0.98°C). Geckos in the dry season preferred temperatures 0.77°C lower than those in the wet season (95% CI=0.36–1.19°C; Fig. 2A). As with body condition, however, the species×season analysis indicated that the seasonal effect on *T*_pref_ was restricted to the three species sampled at Kidman Springs (*G. gemina*, *G. koira* and *G. nana* [KS]), with no corresponding effect of season in the species sampled at Litchfield (Fig. 2B, Table S6).

### Evaporative water loss

**Fig. 3.**
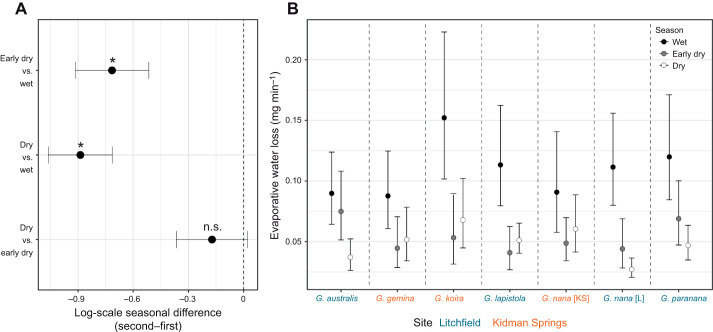
**Seasonal variation in evaporative water loss (EWL).** (A) Pairwise contrasts of EWL among the three seasons (posterior mean±95% CI), showing lower rates in the early dry and dry seasons compared to the wet season. (B) Predicted EWL rates for each species by season (posterior mean±95% CI). Coloured text indicates the two sampling sites: the more mesic Litchfield National Park and the more arid Kidman Springs. All species show at least one significant difference between the wet season and one or both dry seasons at *P*<0.05. All values and effect sizes are adjusted to the mean body condition and surface area (30 cm²) and averaged across sex. See Table 1 for sample size for each species.

Rates of evaporative water loss were positively associated with SA and negatively associated with body condition, and no effect of sex was detected (Table S7). EWL rates increased by an average of 3.43% for every 1 cm^2^ increase in SA (95% CI=2.42–4.40%). All covariate effects were robust to FDR adjustment. After accounting for SA and body condition – and averaging across sex – EWL decreased by 51% between the wet and early dry seasons (95% CI=40.3–60%) and by 59% between the wet and dry seasons (95% CI=51.1–65.4%), with no significant difference between the early dry and dry seasons (Fig. 3A; Table S8). Although the extent of the seasonal changes in EWL varied among species, seasonal effects were broadly consistent (Table S8), with higher rates of EWL in the wet season for every species (Fig. 3B).

## DISCUSSION

Seasonal acclimatisation enables individuals to adjust physiological parameters to better cope with changing environmental conditions, a crucial aspect of physiological ecology observed in some reptiles (Basson and Clusella-Trullas, 2015; Blamires and Christian, 1999; Christian et al., 1983; Clusella-Trullas and Chown, 2014; Dubiner et al., 2023). Despite the importance of this adaptability (Berg et al., 2017; Christian et al., 1999a,b, 2024), few studies have focused on the ability of reptiles to exhibit seasonal flexibility in EWL (Rozen-Rechels et al., 2019a,b; Weaver et al., 2023). Although there is a direct mechanistic relationship between body temperature and EWL (Kearney et al., 2018; Mautz, 1982; Rozen-Rechels et al., 2019a,b, 2021), we found differences in the seasonal patterns of these parameters among species. With respect to seasonal thermal preference, we found that whereas four species from a more thermally stable, mesic site showed no seasonal differences in thermal preference, three species from a semi-arid, thermally variable location exhibited significantly lower thermal preference during the cooler dry season. With respect to seasonal EWL, we found that dry season rates were lower (by 34–76%) compared with the wet season, indicating a substantial acclimatisation response, with rates falling sharply from the wet to early dry season, then either remaining low or decreasing further later in the dry season.

Compared with other lizards, the *T*_pref_ values of geckos are low, but this is not simply a passive consequence of nocturnality (Meiri, 2019). If there is heterogeneity in the thermal environment (Muñoz et al., 2014), nocturnal geckos can regulate body temperatures during the day through selection of retreat sites (Kearney and Predavec, 2000; Shah et al., 2004), which can change seasonally (Kearney, 2002). For some *Gehyra* spp., behavioural thermoregulation is a high priority when thermal conditions are sub-optimal (Grimm-Seyfarth et al., 2018). Although *T*_pref_ did not differ between seasons for the four *Gehyra* species from the mesic site with relatively stable seasonal temperatures (Litchfield), all three species from the semi-arid site with a greater range of seasonal temperatures (Kidman Springs) had significantly lower *T*_pref_ in the dry season. Lower body temperatures result in both energy conservation (Christian et al., 1983; Christian and Bedford, 1995, 1996; Clusella-Trullas and Chown, 2014) and decreased EWL (Malvin and Wood, 1991). Although samples from additional sites would be needed to establish a definitive relationship, the different patterns between the two sites (and the results from the two populations of *G. nana*) are consistent with there being a correlation between the capacity for acclimatisation and variability in environmental conditions (Christian et al., 2024; Muñoz and Bodensteiner, 2019).

There were strong and significant reductions in EWL from wet to dry season in all the species in this study. This result aligns with our prediction and the measurements of other reptiles from the seasonal tropics (Blamires and Christian, 1999; Day et al., 2025). The significant difference in EWL between wet and early dry seasons for most species suggests that EWL changes rapidly in response to reduced humidity. In recent laboratory experiments, some North American lizards decreased EWL in a matter of days after exposure to constant dry conditions (Weaver et al., 2023). However, under field conditions, the transitional period would probably be influenced by local conditions and variable weather events. Metabolic rates may also have been depressed in the dry season, as has been shown in other lizards from the seasonal tropics (Christian et al., 1999a,b, 2024), and this would have resulted in lower respiratory water loss. However, given that cutaneous EWL accounts for approximately 70% of the total in lizards (Blamires and Christian, 1999; Mautz, 1982), the large seasonal changes would necessarily involve cutaneous changes, which is consistent with laboratory studies (Kattan and Lillywhite, 1989; Weaver et al., 2023). In laboratory acclimation experiments in other lizards, decreased EWL was attributed to changes in skin permeability caused by lipid redistribution (Kattan and Lillywhite, 1989; Dubiner et al., 2025). Inspection of EWL rates indicates a greater range of values among individuals in the wet season compared with dry season rates, suggesting that the dry season EWL may be approaching the minimum skin permeability that the geckos can physiologically achieve. In the wet season, the release from hydric stress results in higher EWL generally, but differences in local conditions may result in the observed variability among individuals during this season. Acclimatisation of EWL apparently provides greater benefits than fixing EWL at a low rate year-round, suggesting some unquantified cost of maintaining low EWL, possibly related to the biochemical synthesis of water-conserving barriers (Kattan and Lillywhite, 1989), as has been shown in desert scorpions (Toolson and Hadley, 1979), ants (Menzel et al., 2018), fruit flies (Stinziano et al., 2015) and desert birds (Haugen et al., 2003). Research into the mechanics of EWL reduction, including the energetic consequences, is needed.

Both thermal and metabolic acclimatisation in the seasonal tropics have been well documented (Berg et al., 2017; Christian and Bedford, 1995, 1996; Christian et al., 1983, 1999a,b, 2024) and there is growing evidence with respect to EWL acclimatisation in lizards from wet–dry tropical regions from four families. Seasonal acclimatisation has now been documented in 14 species of lizards from the wet–dry tropics of Australia including one species from the family Agamidae, *Tropicagama temporalis* (Blamires and Christian, 1999), eight species of Gekkonidae (the six *Gehyra* species reported here, *Heteronotia binoei* and *Hemidactylus frenatus*; Day et al., 2025), one species of Diplodactylidae (*Amalosia rhombifer*; Day et al., 2025) and four species of Scincidae (*Cryptoblepharus metallicus*, *Carlia rufilatus*, *Ctenotus robustus* and *Ctenotus essingtonii*; Rachmansah, 2025). Arguments that the relative stability in environmental conditions in some tropical regions negates the need for physiological acclimatisation (Sun et al., 2022) have been over-generalized to include the entire tropics despite there being substantial evidence for a link between the physiological capacity for acclimatisation and the wet–dry tropical climate (Christian et al., 2024). The general lack of attention to EWL acclimatisation (Weaver et al., 2022, 2023) means that little is known about seasonal acclimatisation of EWL of reptiles in the field from the wet tropics and other climatic zones. A temperate desert species, *Gambelia sila*, did not show a change in cutaneous water loss across its activity period despite environmental conditions becoming progressively hotter and drier (Weaver et al., 2024). To date, all 14 lizard species from the seasonal tropics that have been measured across seasons have shown significant acclimatisation to EWL, and there are currently no ‘field’ data (measurements taken directly from wild-caught individuals) demonstrating acclimatisation in EWL from other climatic regions. The seasonal tropics are different from the aseasonal (wet) tropics with respect to water availability across the year and the prolonged dry season also results in substantial seasonal differences in food availability (Griffiths and Christian, 1996). Furthermore, the seasonal tropics are different from temperate regions with respect to there being a relatively stable thermal environment across the year. Inactivity during winter conditions makes it difficult to find reptiles (AlRashidi et al., 2025), and the reduced metabolic rates and the protective environments in hibernacula may obviate the need for acclimatisation in EWL. Thus, we are left with the question: do the distinctive environmental conditions of relatively high and stable environmental temperatures coupled with prolonged dry conditions mean that seasonal acclimatisation of EWL is an adaptation that is primarily associated with the seasonal tropics, or is it simply the case that we do not yet know enough about seasonal EWL in lizards from other climatic regions? Geographic bias in our knowledge across climatic regions has been documented with respect to both EWL (Le Gilliard et al., 2021a,b) and physiological plasticity (Seebacher et al., 2015). Substantial metabolic depression in lizards that are nevertheless above ground and active (albeit less active than during the wet season) has, to our knowledge, only been found during the dry season in the seasonal tropics (Berg et al., 2017; Christian et al., 1999a, 2024; Milsom et al., 2008) and it is possible that seasonal acclimatisation of EWL in lizards may also be a characteristic associated with the climatic conditions unique to the seasonal tropics. Comparisons of EWL between active and inactive seasons for temperate-zone lizards would be instructive, but logistically challenging.

Several lizards from the wet–dry tropics are known to have lower thermal preferences and lower field body temperatures in the dry season relative to the wet season, which results in energetic savings when food availability is reduced (Christian and Bedford, 1995, 1996; Christian and Weavers, 1996; Christian et al., 1998, 1999b, 2003). The species that do not follow this pattern, being those that live near water and abundant food resources (*Varanus mertensi*, *Varanus chlorostigma* (formerly *Varanus indicus*) and some populations of *Varanus panoptes*), reinforce the conclusion that the conservation of energy and water are the benefits of the seasonal changes (Christian and Weavers, 1996; Smith et al., 2008). The mechanism driving differences in seasonal thermal preferences among *Gehyra* spp. in this study is unknown. Some forest-dwelling lizards in the aseasonal tropics apparently lack the capacity for thermal acclimation (Huey et al., 2009), but we do not know if the absence of seasonal acclimatisation of preferred body temperature in the geckos from the relatively thermally stable site (Litchfield) indicates that they lack the genetic capacity for thermal acclimatisation of thermal preference. It would be instructive to determine whether other aspects of the thermal biology (i.e. critical thermal temperatures) of the Litchfield geckos are similarly unresponsive to seasonal patterns. If so, these populations may be endangered if appropriate thermal refugia become scarce or are no longer available because of climate change. However, the ability to change EWL in response to environmental conditions would benefit these geckos during prolonged drought if climate change disrupts the current rainfall and humidity patterns. In fact, the physiological adaptations of a taxonomically diverse range of lizards in the wet-dry tropics to the extreme shifts in water (and consequently food) availability (Christian et al., 1999a; Day et al., 2025; Skelton et al., 2025) may represent pre-adaptations to climate change that are not available to lizards from the wet tropics (Christian et al., 2024).

## Supplementary Material

10.1242/jexbio.250797_sup1 Supplementary information

Table S1. Data from measurements of evaporative water loss (EWL) from six species of *Gehyra* geckos collected during three seasons from two sites in tropical Australia. Statistical results from these data are shown in Fig. 3.

Table S2. Data from measurements of preferred body temperature (T_pref_) from six species of *Gehyra* geckos collected during two seasons from two sites in tropical Australia. Statistical results from these data are shown in Fig. 2.
