# Supplementary material for: Acclimatisation to tropical seasons: hydric and thermal physiology in Gehyra geckos
Source: J Exp Biol. 2026 Mar 13;229(5):jeb250797. doi: 10.1242/jeb.250797 (PMC13006520; doi:10.1242/jeb.250797)
Supplement: Supplementary information [file jexbio-229-250797-s1.pdf]

Table S1. Data from measurements of evaporative water loss (EWL) from six species of *Gehyra* geckos collected during three seasons from two sites in tropical Australia. Statistical results from these data are shown in Fig. 3.

Available for download at
https://journals.biologists.com/jeb/article-lookup/doi/10.1242/jeb.250797#supplementary-data

Table S2. Data from measurements of preferred body temperature (T_{pref}) from six species of *Gehyra* geckos collected during two seasons from two sites in tropical Australia. Statistical results from these data are shown in Fig. 2.

Available for download at
https://journals.biologists.com/jeb/article-lookup/doi/10.1242/jeb.250797#supplementary-data

Table S3. Results of the phylogenetically informed analysis of season on body condition using MCMCglmm, with log-transformed mass as the response variable and phylogenetic covariation included as a random effect

Term	Mean	LowerCI	UpperCI	pMCMC	FDR –adjusted pMCMC
log(SVL)	2.9432	2.7913	3.0982	**0.0000**	**0.0001**
early dry-wet	0.9698	0.9135	1.0288	0.2965	0.5923
dry-wet	0.9432	0.8975	0.9900	**0.0210**	0.1008
dry-early dry	0.9726	0.9206	1.0269	0.3260	0.5923

The table reports the effects of log-transformed snout–vent length (SVL) and the three pairwise seasonal contrasts. Coefficients are given as posterior means with 95% credible intervals. For log(SVL), coefficients indicate the percentage increase in mass per 1% increase in SVL, while for seasonal contrasts they indicate the proportional change in mass between the first and second season (negative values = decrease in the second season). Both raw and FDR-adjusted pMCMC values are reported

Journal of Experimental Biology • Supplementary information

Table S4. Results of the MCMCglmm analysis of season on body condition, including the species × season interaction but excluding phylogenetic covariance

Species	Contrast	Contrast mean	Lower CI	Upper CI	pMCMC	FDR –adjusted pMCMC
G. australis (L)	early dry-wet	1.0958	0.9541	1.0481	0.1805	0.4332
	dry-wet	1.0352	0.9106	1.0982	0.5870	0.7415
	dry-early dry	0.9447	0.8257	1.2112	0.4095	0.6143
G. gemina (KS)	early dry-wet	0.8870	0.7615	1.3131	0.1195	0.3187
	dry-wet	0.7988	0.6875	1.4546	**0.0040**	**0.0320**
	dry-early dry	0.9005	0.7625	1.3114	0.2130	0.4647
G. koira (KS)	early dry-wet	0.8412	0.7064	1.4157	0.0505	0.2020
	dry-wet	0.8288	0.7189	1.3910	**0.0125**	0.0750
	dry-early dry	0.9852	0.8167	1.2244	0.8595	0.8969
G. lapistola (L)	early dry-wet	1.1369	0.9818	1.0186	0.0825	0.2475
	dry-wet	1.0032	0.8951	1.1172	0.9490	0.9490
	dry-early dry	0.8824	0.7789	1.2839	0.0595	0.2040
G. nana (KS)	early dry-wet	0.7233	0.6267	1.5957	**0.0000**	**0.0007**
	dry-wet	0.7726	0.6659	1.5017	**0.0020**	**0.0240**
	dry-early dry	1.0682	0.9334	1.0713	0.3455	0.5923
G. nana (L)	early dry-wet	1.0642	0.9172	1.0903	0.3845	0.6143
	dry-wet	1.0256	0.9233	1.0831	0.6580	0.7896
	dry-early dry	0.9637	0.8459	1.1822	0.5870	0.7415
G. paranana (L)	early dry-wet	1.0293	0.8979	1.1137	0.7010	0.8011
	dry-wet	1.0447	0.9172	1.0903	0.4740	0.6692
	dry-early dry	1.0150	0.8955	1.1167	0.8125	0.8864

Pairwise seasonal contrasts are shown for each species. Coefficients are given as posterior means with 95% credible intervals and indicate the proportional change in mass between the first and second season (negative values = decrease in the second season). Both raw and FDR-adjusted pMCMC values are reported.

Journal of Experimental Biology • Supplementary information

Table S5. Results of the phylogenetically informed MCMCglmm analysis of season on preferred body temperature (Tpref, °C), with phylogenetic covariation included as a random effect

Term	Mean	LowerCI	UpperCI	pMCMC	FDR – adjusted pMCMC
sexM-sexF	−0.5708	−0.9854	−0.1630	**0.0095**	**0.0285**
body-condition	1.2605	−0.2358	2.7363	0.0920	0.1840
dry-wet	−0.7725	−1.1930	−0.3637	**0.0000**	**0.0004**

Table S6. Results of the MCMCglmm analysis of season on preferred body temperature (Tpref, °C), including the species × season interaction but excluding phylogenetic covariance

Species	Contrast	Contrast mean	Lower CI	Upper CI	pMCMC	FDR – adjusted pMCMC
G. australis (L)	dry-wet	0.8859	−0.4177	2.1887	0.1735	0.2974
G. gemina (KS)	dry-wet	−1.2630	−2.2597	−0.2624	**0.0160**	**0.0384**
G. koira (KS)	dry-wet	−2.0366	−3.0726	−1.0055	**0.0005**	**0.0020**
G. lapistola (L)	dry-wet	−0.1118	−0.9706	0.7524	0.8035	0.8765
G. nana (KS)	dry-wet	−1.5239	−2.5086	−0.5500	**0.0005**	**0.0020**
G. nana (L)	dry-wet	−0.2915	−1.2436	0.6329	0.5325	0.7100
G. paranana (L)	dry-wet	−0.6414	−1.8114	0.5442	0.2885	0.4328

Coefficients are given as posterior means with 95% credible intervals and indicate the change in °C between the first and second levels of factors (sex and season) and per unit increase in body condition. Both raw and FDR-adjusted pMCMC values are reported.

Pairwise seasonal contrasts are reported for each species. Coefficients are given as posterior means with 95% credible intervals and indicate the change in °C between the first and second season. Both raw and FDR-adjusted pMCMC values are reported.

Journal of Experimental Biology • Supplementary information

Table S7. Results of phylogenetically informed MCMCglmm analysis of seasonal effects on evaporative water loss (EWL, mg min^{-1}), with phylogenetic covariation included as a random effect

Term	Mean	Lower CI	Upper CI	Mean depression	Lower CI depression	Upper CI depression	pMCMC	FDR – adjusted pMCMC
surface area	1.0343	1.0242	1.0441	NA	NA	NA	**0.0000**	**0.0001**
body-condition	0.5188	0.3231	0.8333	NA	NA	NA	**0.0074**	**0.0187**
sexM-sexF	1.1091	0.9319	1.3181	−10.9063	−31.8059	6.8124	0.2410	0.3182
dry-earlyDry	0.8421	0.6938	1.0215	15.7933	−2.1513	30.6238	0.0812	0.1411
dry-wet	0.4118	0.3458	0.4900	58.8157	51.0027	65.4162	**0.0000**	**0.0001**
earlyDry-wet	0.4891	0.4009	0.5971	51.0915	40.2865	59.9105	**0.0000**	**0.0001**

Coefficients are given as posterior means with 95% credible intervals and represent the change in EWL per unit increase in surface area (SA) and body condition, and the proportional change between the first and second levels of sex and season. For sex and season, the percentage depression in EWL is also reported. Both raw and FDR-adjusted pMCMC values are reported.

Journal of Experimental Biology • Supplementary information

Table S8. Results of the MCMCglmm analysis of season on evaporative water loss (EWL, mg min^{-1}), including the species × season interaction but excluding phylogenetic covariance

Species	Contrast	Contrast mean	Lower CI	Upper CI	Mean depression	Lower CI depression	Upper CI depression	pMCMC	FDR – adjusted pMCMC
G. australis (L)	early dry-wet	0.8297	0.5160	1.3446	17.0330	−34.4583	48.3990	0.4415	0.4857
	dry-wet	0.4132	0.2660	0.6513	58.6820	34.8743	73.3968	**0.0010**	**0.0033**
	dry-early dry	0.4980	0.3057	0.8035	50.1995	19.6466	69.4339	**0.0055**	**0.0151**
G. gemina (KS)	early dry-wet	0.5056	0.2939	0.8830	49.4442	11.7002	70.6100	**0.0190**	**0.0418**
	dry-wet	0.5868	0.3446	1.0022	41.3188	−0.2159	65.5382	0.0520	0.0953
	dry-early dry	1.1607	0.6530	2.0458	−16.0721	−104.5844	34.7027	0.5970	0.6355
G. koira (KS)	early dry-wet	0.3481	0.1847	0.6418	65.1868	35.8245	81.5300	**0.0015**	**0.0045**
	dry-wet	0.4498	0.2656	0.7511	55.0198	24.8872	73.4395	**0.0000**	**0.0001**
	dry-early dry	1.2920	0.6881	2.4259	−29.2047	−142.5871	31.1892	0.4375	0.4857
G. lapistola (L)	early dry-wet	0.3606	0.2102	0.6084	63.9424	39.1611	78.9824	**0.0000**	**0.0001**
	dry-wet	0.4472	0.3032	0.6546	55.2814	34.5396	69.6808	**0.0005**	**0.0018**
	dry-early dry	1.2402	0.7888	1.9728	−24.0200	−97.2849	21.1230	0.3425	0.4347
G. nana (KS)	early dry-wet	0.5390	0.3147	0.9085	46.0990	9.1528	68.5293	**0.0175**	**0.0413**
	dry-wet	0.6627	0.3838	1.1148	33.7273	−11.4841	61.6194	0.1380	0.2169
	dry-early dry	1.2295	0.7496	1.9701	−22.9526	−97.0069	25.0416	0.4000	0.4857
G. nana (L)	early dry-wet	0.3954	0.2393	0.6501	60.4558	34.9906	76.0653	**0.0000**	**0.0001**
	dry-wet	0.2435	0.1681	0.3565	75.6545	64.3491	83.1927	**0.0000**	**0.0001**
	dry-early dry	0.6157	0.3803	0.9908	38.4346	0.9157	61.9706	**0.0465**	0.0903
G. paranana (L)	early dry-wet	0.5772	0.3463	0.9457	42.2806	5.4268	65.3672	**0.0315**	0.0650
	dry-wet	0.3926	0.2554	0.6109	60.7397	38.9092	74.4642	**0.0000**	**0.0001**
	dry-early dry	0.6802	0.4312	1.0869	31.9809	−8.6881	56.8771	0.1005	0.1658

Pairwise seasonal contrasts are reported for each species. Coefficients are given as posterior means with 95% credible intervals and indicate the change in EWL between the first and second season. For sex and season, the percentage depression in EWL is also reported. Both raw and FDR-adjusted pMCMC values are reported.

Journal of Experimental Biology • Supplementary information
